# Integrative analysis of green ash phloem transcripts and proteins during an emerald ash borer infestation

**DOI:** 10.1186/s12870-023-04108-y

**Published:** 2023-03-03

**Authors:** Christine C. Chiu, Gervais Pelletier, Juliana Stival Sena, Florence Roux-Dalvai, Julien Prunier, Arnaud Droit, Armand Séguin

**Affiliations:** 1 Laurentian Forestry Centre, Canadian Forest Service, Natural Resources Canada, Quebec City, QC Canada; 2 CHU de Québec–Laval University Research Centre, Université Laval, QC Quebec City, Canada

**Keywords:** Emerald ash borer, *Fraxinus*, Plant defense, Transcriptomics, Proteomics, Plant–insect interactions, Forest

## Abstract

**Background:**

Emerald ash borer (*Agrilus planipennis*; EAB) is an Asian insect species that has been invasive to North America for 20 years. During this time, the emerald ash borer has killed tens of millions of American ash (*Fraxinus* spp) trees. Understanding the inherent defenses of susceptible American ash trees will provide information to breed new resistant varieties of ash trees.

**Results:**

We have performed RNA-seq on naturally infested green ash (*F. pennsylvanica*) trees at low, medium and high levels of increasing EAB infestation and proteomics on low and high levels of EAB infestation. Most significant transcript changes we detected occurred between the comparison of medium and high levels of EAB infestation, indicating that the tree is not responding to EAB until it is highly infested. Our integrative analysis of the RNA-Seq and proteomics data identified 14 proteins and 4 transcripts that contribute most to the difference between highly infested and low infested trees.

**Conclusions:**

The putative functions of these transcripts and proteins suggests roles of phenylpropanoid biosynthesis and oxidation, chitinase activity, pectinesterase activity, strigolactone signaling, and protein turnover.

**Supplementary Information:**

The online version contains supplementary material available at 10.1186/s12870-023-04108-y.

## Background

The emerald ash borer (*Agrilus planipennis*; EAB) is a wood-boring insect that is invasive to North America. Since its detection in Michigan in 2002, EAB and has killed tens of millions of ash trees (*Fraxinus* spp) across eastern Canada and the United States [1]. EAB generally only colonizes stressed or dying ash trees within its native range of east Asia, including areas of the countries of China, Japan, Korea, Mongolia and Russia [2]. Asian *Fraxinus* species that share a coevolutionary history with EAB, such as Manchurian ash (*F. mandshurica*), are more resistant to EAB colonization than their North American counterparts. In contrast, susceptible North American ash trees suffer 58 -100% mortality in the years following EAB colonization, depending on the ash species and forest composition [3–5]. Amongst them, green ash (*Fraxinus pennsylvanica*), white ash (*Fraxinus americana*), and black ash (*Fraxinus nigra*) are the major ash species under threat by EAB in North America [6].

The life cycle of EAB can last 1–2 years, depending on the environmental conditions [7, 8]. EAB larvae create serpentine feeding galleries in the phloem and xylem tissue that disrupt the transport of water and nutrients of the host tree [9]. After adult EAB have emerged from characteristic "D" shaped exit holes in the bark, they feed on the leaves in the upper bole of the tree, mate, and then deposit their eggs into bark crevices [7]. Studies on resistance mechanisms of ash trees to the EAB have focused on traits that influence oviposition preference or that limit larval feeding in the tree [8].

Identifying resistance traits against EAB in ash species is key to breeding new resistant varieties of ash. Some strategies of elucidating EAB-resistant traits compare resistant Manchurian ash to susceptible ash species such as green, white, black or European ash. Multiple analyses of the phenolic profiles of different ash species overall indicate that Manchurian ash employs lignans and a specific derivative of coumarin as a chemical defense against EAB larvae [9–11]. Additionally, phenolics can be further oxidized by the activity of polyphenol oxidases and peroxidases to oxidized polyphenols and phenoxyl radicals, which create an oxidative environment for the developing larvae [12, 13] Differences in the activity of these enzymes could account for the faster browning reaction observed in Manchurian ash than in white or green ash [9]. In a study of 26 *Fraxinus* taxa, several genes related to phenylpropanoid biosynthesis pathway, herbivore recognition, defense signalling or programmed cell death were identified as potential resistance genes in EAB-resistant ash species [14].

Research indicates that even susceptible ash species do have potentially EAB defensive traits. A small percentage (< 1%) of green and white ash survive or escape EAB attack [15, 16]. Previous studies have indicated that the tree may not be recognizing or responding quickly enough to EAB cues of infestation. Green, white, and black ash trees primed with the elicitor methyl jasmonate have a higher resistance to EAB colonization than those that were not primed, and produced more of the plant defense compounds lignin, trypsin inhibitors and verbascoside [17]. This seems to indicate that an effective defense response against EAB does exists in these American ash species, and that the effectiveness may depend on the timing of the response.

Further characterization of the transcript and protein changes that occur during the early stages of EAB colonization would clarify the relationship between the timing of the defense response and ash borer resistance. Previous ash transcriptomics studies [18, 19], the genetic linkage map of green ash [20] and the genomes of European common ash (*F. excelsior*) [21] and green ash (*F. pennsylvanica*) [22] provide genomic resources to build upon.

In 2012, the presence of EAB was confirmed in the environs of the city of Montreal [23]. During subsequent years of monitoring for the eventual spread of the insect, EAB was detected in the surrounding regions of Repentigny, Lavaltrie and Berthierville [23]. In this environment of the increasing invasion of EAB, we identified ash trees with low, medium and high levels of infestation.

We have conducted RNA-Seq and proteomics on naturally infested green ash trees at low, medium and high levels of EAB infestation. Our objective was to identify transcripts and proteins that are involved in the defense response of green ash during the progression of emerald ash borer infestation. We have integrated the analysis of the green ash transcriptomics and proteomics to identify a unique set of genes linked to three different levels of increasing EAB infestation of the tree.

## Results

We identified urban ash trees in different municipalities northeast of Montreal showing low, moderate or high levels of EAB infestation (Table 1) that we investigated at both the transcriptomic and proteomics levels. Seventeen RNA libraries were constructed for transcript quantification and a total of ~ 297 million reads were obtained with an average of 20 million reads per library. In average 78% of reads per library were mapped in pairs, 13% mapped in broken pairs and 8% was not mapped (Table S1).

**Table 1:** Trees sampled for transcriptomic and proteomic analysis. Infested trees were selected from the different municipalities northeast of Montreal. Selected trees were placed into an infestation category of low, medium or high based on EAB trap catches and ash canopy condition

Tree #	Infestation Category	Diameter at Breast Height	Number of EAB in Trap	Ash canopy condition rating	Location	Used For Transcriptomics (T) or Proteomics (P)
		(cm)		(1–5)		
**1**	High	86.6	277	4	Laval	T & P
**2**	High	28.2	43	4	Laval	T & P
**3**	High	66.8	669	4	Laval	T & P
**4**	High	40.9	90	3	Laval	T
**5**	High	76.2	233	4	Laval	T & P
**6**	Medium	28	29	1	Repentigny	T
**7**	Medium	29.3	85	1	Repentigny	T
**8**	Medium	31.6	77	1	Repentigny	T
**9**	Medium	30	81	2	Repentigny	T
**10**	Medium	31.1	64	2	Repentigny	T
**14**	Low	26.5	12	1	Lavaltrie	T & P
**15**	Low	36.3	2	1	Lavaltrie	T & P
**17**	Low	52	0	1	Berthierville	T & P
**18**	Low	59.9	3	3	Berthierville	T & P
**19**	Low	38.2	1	2	Berthierville	T

**Fig. 1:**
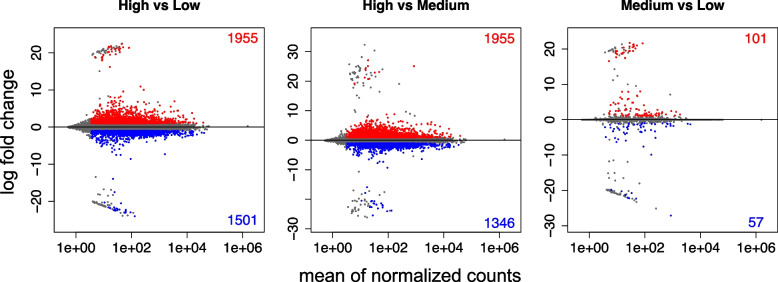
MA plots showing the log fold change of transcripts when comparing a) high vs low infestation, b) high vs medium infestation, c) medium vs low infestation. Significantly up-regulated genes are shown in red and significantly down-regulated genes are shown in blue. The number of up-regulated transcripts are shown in the upper right corner and the number of down-regulated transcripts are shown in the lower right corner of each plot

We found 93,810 transcripts with a non-zero read count in our RNA-Seq experiment representing 87% of the green ash transcriptome. We analyzed these transcripts for differential expression between the infestation levels. Between low and medium infestation, 101 (0.1%) transcripts were significantly up-regulated and 57 (0.06%) significantly down-regulated (Fig. 1). Between medium and high infestation, 1955 (2.1%) transcripts were significantly up-regulated and 1346 (1.4%) significantly down-regulated. Between low and high infestation, 1955 (2.1%) transcripts were significantly up-regulated and 1501 (1.6%) down-regulated. In addition, 1280 (1.4%) and 19,445 (21%) of transcripts were respectively shown to have an outlying number of high and low counts and were thus eliminated from the differential expression analysis. The putative functions of all differentially expressed transcripts can be found in Tables S2-S4.

**Fig. 2:**
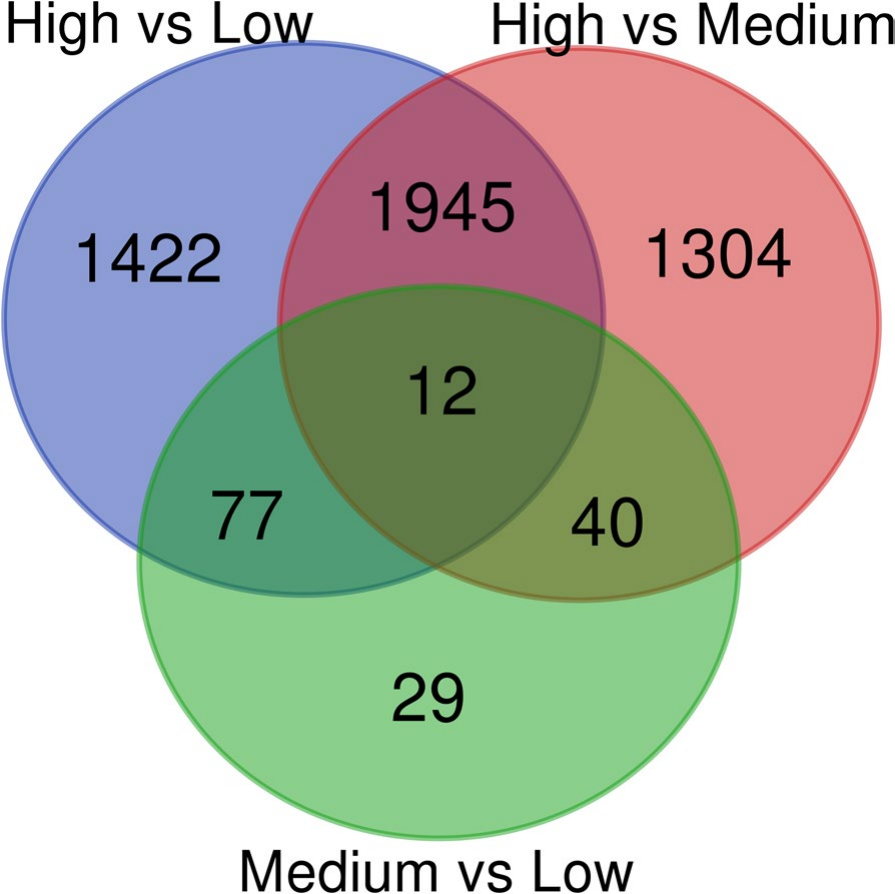
Venn diagram showing the number of differentially expressed transcripts found in comparisons of high vs low infestation (High-Low), high vs medium infestation (High-Medium), and medium vs low infestation (Medium–Low). Overlapping areas indicate the number transcripts that were found to be differentially expressed across all the overlapping groups. For example, 12 transcripts were found to be differentially expressed in all the comparisons

We used quantitative PCR (qPCR) to independently validate the expression of 12 differentially expressed transcripts. Correlation of the qPCR and RNA-Seq expression showed consistency between the two methods, with R values of 0.79–98 (Fig. S1). Of the differential expressed transcripts found between each infestation level, 29 were unique to medium–low, 1304 were unique to high-medium, and 1422 were unique to high-low (Fig. 2). The medium–low and high-medium shared 40 differentially expressed transcripts, the medium–low and high-low shared 77 transcripts. The high-low and high-medium shared 1945 transcripts. Twelve transcripts were found to be differential expressed in all comparisons of infested levels.

**Fig. 3:**
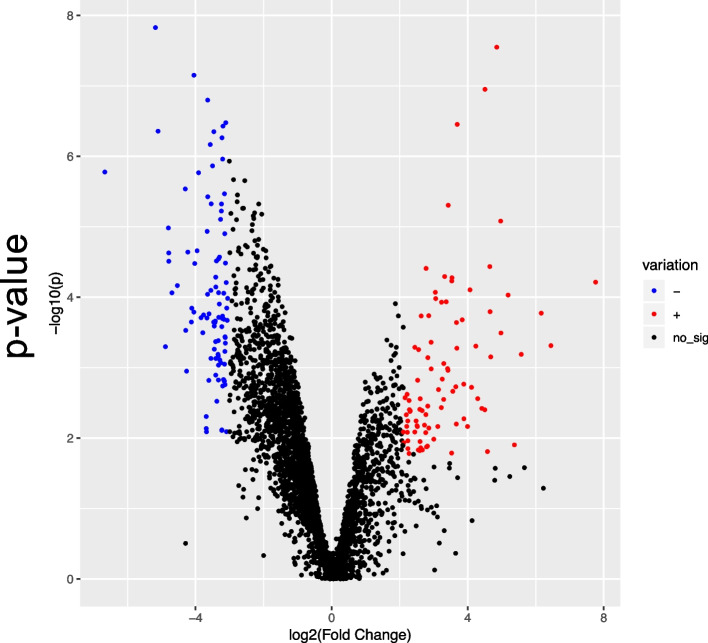
Volcano plot of proteomics of low versus highly infested samples. Proteins in blue are under-represented in infested trees (*p* < 0.05, z < 1.96). Proteins in red are over-represented in infested trees (*p* < 0.05, z < 1.96). Proteins in black are not significantly different between low and highly infested trees

We quantified protein expression in eight of the fifteen ash trees used in our transcript analysis. These trees represented low or high infestation levels (Table 1). Proteomics detected 3912 proteins, 3888 of which had a corresponding transcript found in the RNA-Seq experiments. 188 (4.8%) of proteins were found to be significantly differentially expressed proteins, with 93 (2.3%) up-regulated and 95 (2.4%) down-regulated (Fig. 3). The putative functions of all differentially expressed proteins can be found in Table S5.

**Fig. 4:**
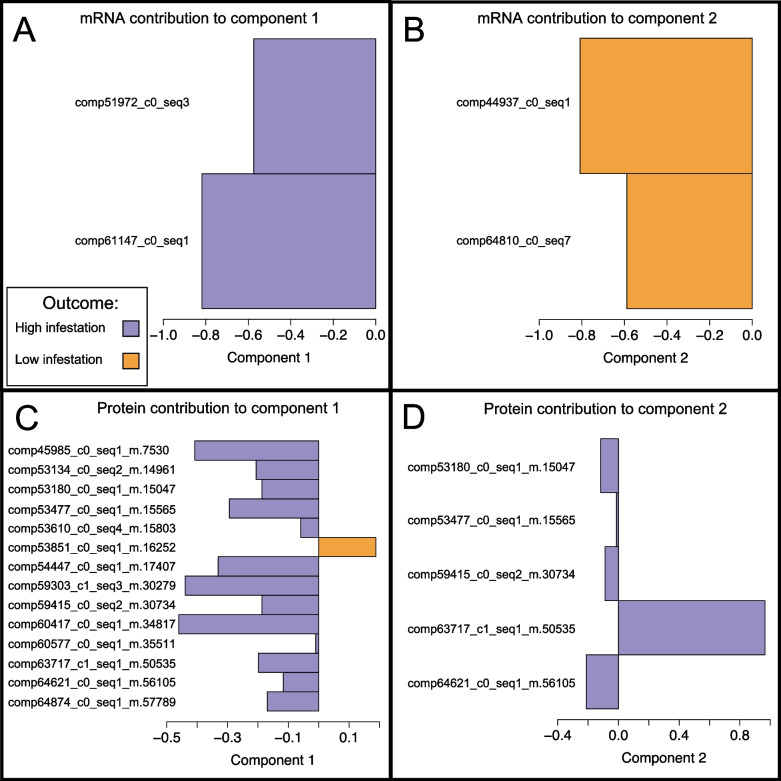
Loading plots of genes identified as the molecular signature. Plots show the maximal mean of **A**: mRNA contribution to component 1, **B** mRNA contribution to component 2, **C** protein contribution to component 1, **D** protein contribution to component 2 of the multivariate model. Colour indicates the outcome that the selected gene contributes towards, high (purple) or low (orange) infestation

**Fig. 5:**
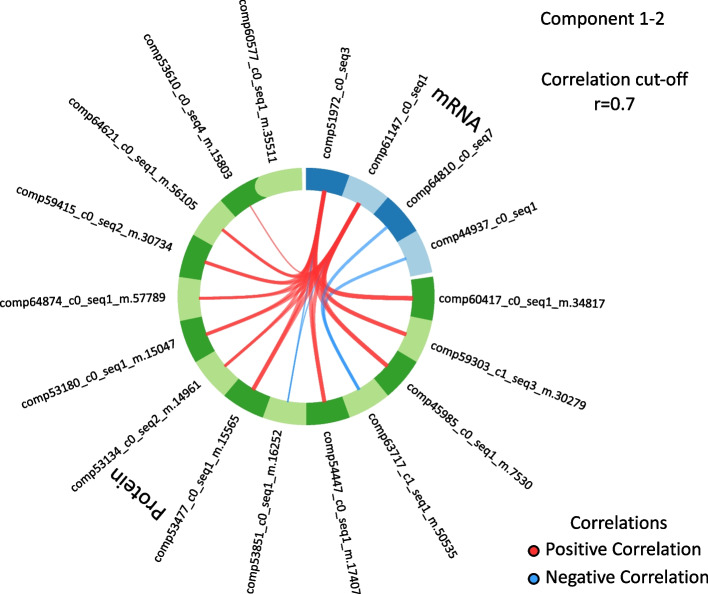
Circos plot showing positive (red) or negative (blue) correlation between selected genes

Our integrative analysis of the RNA-Seq and proteomics data identified 14 proteins and 4 transcripts that contribute most to the difference between highly infested and low infested trees. Of this subset, 13 proteins and 2 transcripts are present in the highly infested state and 1 protein and 2 transcripts are present in the low infested state (Figs. 4 and 5).

The putative functions of proteins and transcripts that contribute to the highly infested state are two dirigent proteins, two chitinases, two peroxidases, a multicopper oxidase, a beta-glucosidase, a pectinesterase, a stringolactone esterase, a transketolase, a caffeoyl-CoA O-methyltransferase, a kynurenine formamidase, an aspartyl aminopeptidase, and a late embryogenesis abundant D 29-like protein (Table 2). The putative functions of proteins and transcripts that contribute to the low infested state are a phosoprotein, a F-box protein and an unknown transcript (Table 2).

**Table 2:** Proposed function of genes selected as the molecular signature by integrative analysis of the transcripts and proteins. Component indicates whether the gene contributes to the component 1 or 2 of the multivariate model. Transcript/Protein indicate either the transcript or protein contributes to the Outcome: high or low infestation. The Top Blast hit and description were obtained from the plant protein database. Gene ontology (GO) annotation and Enzyme activity were obtained from the GO database using Blast2Go.^a ^denotes proteins/transcripts that were also found to be differentially expressed in Lane et al. 2016 [19]

Component	Transcript/Protein	Outcome	Transcript ID	Top Blast hit	Top Blast hit description	Go Annotation	Enzyme activity
1	Protein	High	comp45985_c0_seq1	XP_0228429190.1	probable caffeoyl-CoA O-methyltransferase At4g26220	P: methylation	Caffeoyl-CoA O-methyltransferase
1	Protein	High	comp53134_c0_seq2^a^	XP_0228792430.1	transketolase, chloroplastic-like	P: pentose-phosphate shunt	Transketolase; Formaldehyde transketolase
1	Protein	High	comp53610_c0_seq4	XP_0228935600.1	cationic peroxidase 1-like isoform X2	P: response to oxidative stress	Peroxidase
1	Protein	High	comp54447_c0_seq1	XP_0228534080.1	dirigent protein 22-like	C: apoplast	
1	Protein	High	comp59303_c1_seq3	XP_0128477040.1	beta-glucosidase 40-like	P: carbohydrate metabolic process	Beta-glucosidase
1	Protein	High	comp60417_c0_seq1^a^	XP_0150624040.1	kynurenine formamidase isoform X1	P: tryptophan catabolic process to kynurenine	Arylformamidase
1	Protein	High	comp60577_c0_seq1^a^	XP_0228728610.1	acidic endochitinase-like	P: carbohydrate metabolic process	Chitinase
1	Protein	High	comp64874_c0_seq1	XP_0228522760.1	pectinesterase-like isoform X2	P: pectin catabolic process	Pectinesterase; Carboxylesterase
1	Protein	Low	comp53851_c0_seq1^a^	XP_0228443000.1	phosphoprotein ECPP44-like	P: response to water	
1 & 2	Protein	High	comp53180_c0_seq1	XP_0228703950.1	late embryogenesis abundant protein D-29-like		
1 & 2	Protein	High	comp53477_c0_seq1^a^	XP_0228639540.1	dirigent protein 22-like	C: extracellular region	
1 & 2	Protein	High	comp59415_c0_seq2^a^	XP_0228768020.1	peroxidase 52-like	P: response to oxidative stress;	Peroxidase
1 & 2	Protein	High	comp63717_c1_seq1	XP_0228899110.1	probable aspartyl aminopeptidase	P: chaperone-mediated protein folding	Acting on peptide bonds (peptidases)
1 & 2	Protein	High	comp64621_c0_seq1	XP_0228820390.1	multicopper oxidase LPR2-like	P: oxidation–reduction process	Bilirubin oxidase
1	Transcript	High	comp51972_c0_seq3^a^	XP_0228440310.1	probable strigolactone esterase DAD2	P: secondary shoot formation	Carboxylesterase
1	Transcript	High	comp61147_c0_seq1^a^	XP_0228728610.1	acidic endochitinase-like	P: carbohydrate metabolic process	Chitinase
2	Transcript	Low	comp44937_c0_seq1	–-NA–-			
2	Transcript	Low	comp64810_c0_seq7	XP_0228725640.1	F-box protein At4g00755-like isoform X1		

We compared the differentially expressed transcripts and proteins found in our study to differentially expressed transcripts identified from a previously generated transcriptome of 107 611 transcripts in *F. pennsylvanica* [19]. We found that 1061 of 3456 differentially expressed transcripts from the high-low infestation comparisons, 1101 of 3306 differentially expressed transcripts from the high-medium comparisons, 30 of 158 differentially expressed transcripts from the medium–low comparisons, and 65 of the 187 of the differentially expressed proteins (Tables S6-S9) were also differentially expressed in *F. pennsylvanica* after eight weeks of EAB feeding in the Lane et al. 2016 [19] experiments. Additionally, 6 of the 14 proteins and 2 of the 4 transcripts identified in our integrative analysis were also reported as differentially expressed transcripts after EAB feeding (Tables 2 and S10) [19].

We compared the differentially expressed transcripts and proteins found in our study to 28 candidate orthologue groups identified as associated to EAB resistance through convergent evolution in an ash species wide study [14]. We found 2 of the 28 orthologue groups *Fraxinus_pennsylvanica_120313_comp54634_c0_seq3* and *Fraxinus_pennsylvanica_120313_comp56704_c0_seq12* had corresponding transcripts in the *F. pennsylvanica* transcriptome that were also identified as differential expressed in our high-low and high-medium comparisons (Table S11). The putative functions of these transcripts are cinnamoyl-CoA reductase-like SNL6 and a probable calcium-binding protein CML21, respectively. None of these candidate genes were identified in our set of differentially expressed proteins or in the results of our integrative analysis of the transcriptomic and proteomics data.

## Discussion

Our RNA seq experiment investigates the transcriptional and proteomics changes in the phloem of naturally infested ash trees during the progression of EAB infestation progresses. Our investigation identified three levels of EAB infestation and compared transcripts and proteins expressed at these stages.

Comparisons between transcripts showed that relatively few of the differentially expressed transcripts were found in low-medium comparisons. This indicates that most of the transcriptional changes in response to EAB occur later during the medium and high infestation stage, supporting previous observations that susceptible green ash may be responding too slowly to EAB infestation to be effective. For example, the application of methyl jasmonate, a derivative of the defense-associated hormone jasmonic acid, increased the accumulation of the plant chemical defenses and decrease EAB colonization in otherwise susceptible green and white ash trees [17]. Interestingly, the two transcripts identified in our analysis *Fraxinus_pennsylvanica_120313_comp54634_c0_seq3* and *Fraxinus_pennsylvanica_120313_comp56704_c0_seq12* that correspond to the EAB resistant orthologue groups identified in [14] were not differentially expressed in medium to low comparison indicating that these important resistance genes may only be activated late in the infestation. The 154 differentially expressed transcripts identified in the medium–low comparison could be investigated further to indicate early transcriptional networks that do respond to EAB in naturally infested green ash.

About one third of the differentially expressed transcripts and proteins, found in our analysis were also found in a previous transcriptomic experiment using artificially introduced EAB larvae on grafted saplings [19]. The overlap of these differentially expressed transcripts and proteins shows some consistency between the different ash populations.

The 14 proteins and 4 transcripts identified from the integrative analysis of the RNA-seq and proteomics experiments point to several processes of plant defense against emerald ash borer. The caffeoyl-CoA O-methyltransferase, two dirigent proteins, and a beta-glucosidase identified in the integrative analysis have putative functions associated with phenylpropanoid biosynthesis. Phenolic compounds, such as lignins, lignans, and more specialized metabolites such as verbascoside produced by phenylpropanoid pathway have been identified in the ash chemical defense response against EAB [9, 10]. Caffeoyl-CoA *O*-methyltransferases methylate hydroxyl groups of monolignols and have a core role in phenylpropanoid biosynthesis. Dirigent proteins mediate stereoselectivity of coniferyl alcohol radical coupling, and thereby direct the flow of precursors in lignin pathway [24]. Beta-glucosidases help regulate the glycosylation of phenolic metabolites by hydrolyzing glycosidic bonds, directing phenolic compounds into different pathways or changing them into a more active form. Beta-glucosidases have also been highlighted in previous chemical, transcriptomic and resistant gene studies of ash [12, 14, 19].

The peroxidase and multicopper oxidase proteins identified in our integrative analysis may be involved in the oxidation of phenolic compounds creating oxidized polyphenols, and phenoxyl radicals. The presence of these reactive oxygenated species in the phloem create a hostile environment for the developing larvae and are associated with higher resistance in Manchurian ash [12, 13].

Endo-chitinases are glycosyl hydrolyses that act on the internal 1,4 linkages of the polymer chitin and degrade it into smaller molecules and are upregulated after herbivory or environmental stress as part of plants' pathogenic response [25]. Chitinase activity was also shown to be higher in resistant Manchurian ash compared to susceptible black ash [12].

Putative functions for two proteins of the integrative analysis are associated with late embryonic proteins. The late embryogenesis abundant D 29-like protein, which is associated with the high infestation state while phosphoprotein ECPP44-like, a dehydrin, is associated with the low infested state. Late embryogenesis proteins are a type of highly hydrophilic glycine-rich protein that function in plant growth and development and response to abiotic stress [26].

Putative functions for two proteins of the integrative analysis are associated with protein turn over. The aspartyl aminopeptidase which is associated with the high infestation state and F-box protein which is associated with the low infested state. A F-box protein was also previously identified as a resistance gene in ash [14].

The putative function of a transcript of the integrative analysis is a probable strigolactone esterase DAD2. Strigolactone esterase DAD2 is involved with the strigolactone perception and signalling [27] while strigolactones are known plant hormones involved in branching, leaf senescence, root development, and plant–microbe interactions.

The putative function of a transcript of the integrative analysis is a pectinesterase. Pectinesterases are enzymes that modify pectin, an important component of the plant cell wall. Plant cell wall remodeling is a known response to abiotic stress [28].

## Conclusions

Altogether our study has identified specific transcripts and proteins that are activated at high levels of infestation. These transcripts and proteins are involved in plant defense processes that are consistent with previous studies of EAB infestation. Our strategy strengthens the characterization of the defense response in green ash and could be integrated to with other genomics studies to identify potential future targets for resistance against EAB. The limited transcriptional response of green ash to low levels of infestation highlights the need for a closer look of the transcriptomics and proteomics of early infestation.

## Methods

### Sample collection

EAB-infested green ash trees were sampled from on June 22, 2017, from five urban park sites, located in the municipalities of Laval, Repentigny, Lavaltrie, and Berthierville (QC, Canada). The location of these sites and the distances between the sites and between sampled trees are shown in Table S12. Permission to collect ash material was granted by private owners, institutions and the Parks and Green Spaces departments of the listed municipalities (Tables 1 and S1).

A green 12 funnel Lindgren trap and a green sticky Prism trap were placed in the upper 1/3 canopy of each tree, on the south or southwest face of each tree that was sampled. Each trap was baited with Z-3-hexenol (Solida: 40SY136) and Z-3-lactone (Solida: 40SY001). These traps were placed in selected trees on June 26, 2017 and trap counts were assessed on July 5, July 27 and August 6, 2017.

Ash canopy condition rating of each sampled tree was assessed based on criteria from Knight et al. 2014 [29]. Ash canopy conditions were given a rating from 1 to 5, where, 1-canopy is full and healthy, 2- canopy has started to lose leaves, 3- canopy has less than 50% dieback, 4- canopy has more than 50% dieback, 5- canopy has no leaves.

Trees were placed into an infestation category of high, medium or low based on total number of EAB catches and the ash canopy condition rating. The high infestation category had EAB trap catches > 40 and an ash canopy rating of 3–5. The medium infestation category had EAB trap catches between 40 and 15 and an ash canopy rating of 1–3. The low infestation category had EAB trap catches between 0 and 15 and an ash canopy rating of 1–3.

All of the trees sampled were located in an urban park environment and were free of other apparent pests.

Phloem and xylem samples were collected from infested trees using a cork borer with a diameter of 5 cm. Trees were sampled at breast height and two samples were taken per tree. These two samples were taken approximately 5 cm apart. Phloem and xylem tissue was removed from the sample and frozen on dry ice in the field. Upon return from the field, the sample was homogenised in liquid nitrogen with mortar and pestle and then stored at at -80 °C for further RNA or protein extraction. The two phloem and xylem samples were ground in liquid nitrogen together as one sample. This study complied with relevant institutional, national, and international guidelines and legislation.

### RNA-Seq analysis

#### RNA extraction and sequencing

Phloem and xylem tissue were homogenised in liquid nitrogen with mortar and pestle and then stored at at -80 °C for further RNA or protein extraction. 100 mg of the homogenized tissue was placed in a frozen 2 mL tube containing a ceramic bead and ground for 60 s at a frequency of 26 1/S with a TissueLyser II (Qiagen, Cat. 85,300). Total RNA was isolated using RNeasy Plant Mini kit (Qiagen, Cat. 74,903) with DNase treatments (RNase-Free DNase Set cat. No. 79254) according manufacturer’s instructions. The RNA was quantified using the Qubit 4 fluorometer (ThermoFisher) with the Qubit RNA BR Assay Kit (ThermoFisher, Cat. Q10211). RNA integrity (RIN) was tested with the Agilent RNA 6000 Nano Kit (Agilent, Cat. 5067–1511) on a 2100 Bioanalyzer instrument (Agilent, Cat. G2939BA) and all samples used for RNA-Seq had a RIN greater than 7.

The Illumina NeoPrep Library Prep System was used to prepare samples from 50 ng of total RNA extraction (Illumina, Documents: 15049720v01, 15049725v03, 15059581v02). TruSeq Standard mRNA Library Prep (Illumina, NP-202–1001) was used with the default indexes adapters A to P. At the last step, each processed sample collected from the library card was analysed for library quality check using a DNA 1000 chip on the 2100 Bioanalyzer (Agilent, Cat. 5067–1504). Finally, each sample was normalized manually at 10 nM and then pooled (5 μL × 16 samples) in one library for the Illumina sequencing platform.

The libraries composed of the 16 samples were sequenced into two lines in Rapid-Run Mode (16 samples/line) in a single flow cell for paired-end 100 bp with an Illumina HiSeq 2500 sequencing system. The samples were sequenced at the Centre Hospitalier de l'Université Laval sequencing platform (Quebec City, Canada).

#### Transcriptome mapping

The raw sequencing reads were trimmed, filtered and processed for a quality check using CLC Genomics Workbench (CLCBio, QIAGEN, http://www.clcbio.com). The adaptors and raw reads with a quality score less than 0.05 (default setting, Phread 13, 95%) were removed.

Trimmed reads remaining as pairs were mapped to the *Fraxinus pennsylvanica* 120,313 transcriptome assembly as reference (downloaded from https://www.hardwoodgenomics.org/) (NCBI Bioproject PRJNA273266) [19] using CLC Genomic Workbench software, version 11.0 (CLCBio, QIAGEN). This transcriptome has 107 611 putative unique transcripts. Total read counts per transcript for each sample were exported from CLC Genomics and used for differential analysis. Raw Illumina sequencing reads and read counts that were generated in our study have been deposited in NCBI's Gene Expression Omnibus, accession number GSE212332.

#### Differential expression analysis

Differential expression analysis was conducted using the DESeq2 package and R version 4.0.5 [30, 31]. Infestation category was set as the design. The log fold change for visualization and ranking was shrunk using ashr [32]. Transcripts with outlying high and low counts were eliminated from the differential expression analysis. Transcripts were considered differentially expressed if they had adjusted *p*-values < 0.05 after log_2_ fold shrinkage and a log_2_ fold change greater than |1|. The adjusted *p*-values used the Benjamin-Hochberg correction to control for the false discovery rate. Venn diagrams for overlapping differentially expressed transcripts were generated using the Venn webtool from Bioinformatics and Evolutionary Genomics, Ghent University (http://bioinformatics.psb.ugent.be/webtools/Venn/).

#### Real-time qPCR validation

To independently verify the results of our RNA-seq experiment, twelve genes were selected based on the dynamic range of transcript level between samples with a high, medium or low infestation rating. Primer design and testing optimized primer dimer, allelic specificity and anneal temperature. After an initial 15 min activation step at 95 °C, 40 cycles of PCR were performed using the following amplification conditions; (94 °C, 5 s; 62 °C to 68 °C, 120 s). Each reaction consisted of 0.6 µM of both forward and reverse primers, 1 ng of cDNA and 1 × Quantitech™ SYBR green mix (Qiagen, Cat. 204,145) in a final volume of 10 µl. Ct values from each target gene were normalized using an average Ct from two reference genes. The fold change was calculated using the 2(ΔCt) method.

### Proteomic analysis

#### Sample preparation prior to mass spectrometry

Proteins were extracted from phloem and xylem samples and prepared for proteomic analysis using the following protocol. 200 mg of the homogenized phloem and tissue samples were added to 500 µL of extraction buffer (0.5% sodium deoxycholate, 50 mM 1,4 dithiotreitol, 1 µM Pepstatin (Thermo Fisher Scientific), 1X Complete Mini Roche (Sigma Aldrich) in 50 mM ammonium bicarbonate) was added to each sample. Mechanical extraction was then performed using a Mixer mill MM400 (Retsh) with three inox beads of two cycles of 2 min at 30 Hz, turning the tube racks 180° between the cycles. Samples were centrifugated at 10 000 × g for 15 min at 4 °C to remove pellet debris. The supernatant was then filtered using a 0.45 µm Centrifugal Filter Membrane (Millipore) at 12 000 × g. Five volumes of acetone at -20 °C was added to the filtered sample and incubated at -20 °C overnight. After centrifugation at 16 000 × g for 15 min at 4 °C, the major part of the supernatant was discarded and the remaining acetone was left evaporated under the fume hood. The pellet was then resuspended with 50µL of 50 mM ammonium bicarbonate and protein concentration was measured using Bradford assay.

For each sample, a volume corresponding to 20 µg of proteins was used for subsequent analysis. Volumes were adjusted to 30 µL using 50 mM ammonium bicarbonate and sodium deoxycholate was added to a final concentration of 1%. The samples were heated at 95 °C for 5 min for protein denaturation. Cysteine disulfide bridges were reduced and alkylated using the following procedure. 1,4 dithiothreitol was added to a final concentration of 0.2 mM and incubated at 37 °C for 30 min. This was followed by the addition of iodoacetamide to a final concentration of 0.8 mM and incubated at 37 °C for 30 min in the dark.

Enzymatic digestion of the protein samples was initiated using 400 ng of trypsin enzyme (Promega), corresponding to an enzyme:protein ratio of 1:50, followed by an incubation at 37 °C overnight. Enzymatic digestion was stopped by acidification using 30µL of 3% acetonitrile, 1% trifluoroacetic acid, 0.5% acetic acid. This step also allowed the precipitation of sodium deoxycholate. Samples were finally centrifugated at 16 000 × g for 5 min and the supernatants were collected. The peptides resulting from trypsin digestion contained in these supernatants were purified on StageTips according to [33] using C18 Empore reverse phase. The samples were finally vacuum dried and stored at -20 °C prior to mass spectrometry analysis.

#### LC–MS/MS analysis

Each sample was resuspended at 0.2 µg/µL with 2% acetonitrile, 0.05%. A volume of 5 µL (equivalent to 1 µg peptides) was then analyzed by liquid chromatography coupled to tandem mass spectrometry (LC-MSMS) using an U3000 RSLCnano chromatographic system (Thermo Fisher Scientific) interfaced with an Orbitrap Fusion mass spectrometer (Thermo Fisher Scientific). The chromatographic separation was done on a reverse phase Acclaim PepMap 100 C18 column (75 µm internal diameter, 3 µm particles and 500 mm length; Thermo Fisher Scientific) using a 5–45% solvent B in 90 min gradient (Solvent A: 5% acetonitrile, 0.1% formic acid; solvent B: 80% acetonitrile, 0.1% formic acid) with a flow rate of 300 nl/min while the mass spectrometer was operating in Data Dependent Acquisition mode using Thermo XCalibur software version 3.0.63. Full scan mass spectra (350 to 1800 m/z) were acquired in the orbitrap at a resolution of 120 000. Internal calibration using lock mass on the m/z 445.12003 siloxane ion was used. Each MS scan was followed by acquisition of fragmentation MSMS spectra of the most intense ions for a total cycle time of 3 s (top speed mode). The selected ions were isolated using the quadrupole analyzer in a window of 1.6 m/z and fragmented by Higher energy Collision induced Dissociation (HCD) with 35% of collision energy. The resulting fragments were detected by the linear ion trap in Rapid scan rate. Dynamic exclusion of previously fragmented peptides was set for a period of 20 s and a tolerance of 10 ppm.

#### Database searching and Label Free Quantification

Spectra were searched against a green ash protein database derived from a transcriptome assembly (https://hardwoodgenomics.org/Transcriptome-assembly/1963024–52,899sequences) using the Andromeda module of MaxQuant software v.1.6.3.43 [34]. Trypsin/P enzyme parameter was selected with two possible missed cleavages. Carbamidomethylation of cysteines was set as fixed modification, methionine oxidation and acetylation of protein N-terminus as variable modifications. Mass search tolerance were 4.5 ppm and 0.6 Da for MS and MS/MS respectively. For validation of identifications, a maximum False Discovery Rate of 0.01 at PSM (Peptide Spectrum Match) and protein levels was used based on a target/decoy search. MaxQuant was also used for Label Free Quantification. The ‘match between runs’ option was enabled with 20 min as alignment time window and 0.7 min as match time window values. Only unique and razor peptides were used for quantification. All other parameters were set at default values. Proteomics data (LC–MS/MS raw files and database search results) are available on the ProteomeXchange data repository under the number PXD037126.

#### Data treatment and statistical analysis related to proteomics

The proteinGroups.txt file generated by MaxQuant was used in R software v 3.4 [31] to perform the following steps. The LFQ intensity values of each protein in each sample were normalized using the median of all LFQ intensity values in each sample. Missing values in the dataset were imputed using a noise value calculated as the first centile of all LFQ intensity values of each sample.

Pairwise analyses were then performed to identify differentially expressed proteins between 2 groups of samples. Only proteins having at least 80% of LFQ intensity values, before missing value imputation, in one of the two groups to compare were considered as quantifiable and only proteins with at least 2 quantified peptides were kept for further analysis. For each protein, a ratio between the two conditions to compare was calculated using the average of protein intensities in all samples of the same group. These ratios were then converted into z-score (*z* = (*x*-*μ*)/*σ* were (*x* = log2(ratio); *μ* = average of all log2(ratios); *σ* = standard deviation of all log2(ratios)) for data centering. A Limma statistical test [35] was performed to determine the probability of variation (*p*-value) of each protein between the two groups. The Benjamini–Hochberg method was used to adjust the *p*-values for multiple testing and thus obtain *q*-values. Proteins with a *q*-value < 0.05 and absolute value of *z*-score |*z*|> 1.96 were considered as significantly differentially expressed between the two groups of samples.

#### Integrative analysis

To identify transcripts and proteins involved in defense response to the emerald ash borer from transcripts and proteins abundances, a sparse partial-least-squares discriminant analysis (sPLS-DA) predicting the attack rating from both the transcriptomics and proteomics data sets was performed. A classification model was built using DIABLO [36] from the Mixomics R package [37]. As numbers of transcripts and proteins to consider for variable selection should be provided, a series of explaining variables numbers (from 2 to 50) was tested using cross-validation for each of transcriptomics and proteomics data sets. The best model, according to the error rate, was kept and variables with high loading weights on each component were extracted. In addition, high correlations between variables from different omics possibly indicating a link were extracted.

## Supplementary Information

**Additional file 1:** **Figure S1.** qPCR validation of RNA seq experiment. Normalized counts of RNA-Seq (blue) compared to Fold change of qPCR results (yellow) of eleven selected genes.

**Additional file 2:** **Figure S2.** Corelation of qPCR validation to RNA seq experiment. Normalize counts of RNA-Seq compared to Fold change of qPCR results of eleven selected genes. *R* value is shown on each chart.

**Additional file 3:** **Table S1.** Total trimmed reads mapped to *F. pennsylvanica *transcriptome.

**Additional file 4: Table S2.** The putative functions of differentially expressed transcripts found in high-low comparisons. **Table S3.** The putative functions of differentially expressed transcripts found in high-medium comparisons. **Table S4.** The putative functions of differentially expressed transcripts found in medium-low comparisons. **Table S5.** The putative functions of differentially expressed proteins found in high-low comparisons. **Table S6.** Differentially expressed transcripts found in the high-low comparison that were also differentially expressed in EAB treatment vs control (TvC) experiment from Lane et al. 2016 [19]. **Table S7.** Differentially expressed transcripts found in the high-medium comparison that were also differentially expressed in EAB treatment vs control (TvC) experiment from Lane et al. 2016 [19]. **Table S8.** Differentially expressed transcripts found in the medium-low comparison that were also differentially expressed in EAB treatment vs control (TvC) experiment from Lane et al. 2016 [19]. **Table S9.** Differentially expressed proteins found in the high-low comparison that were also differentially expressed in EAB treatment vs control (TvC) experiment from Lane et al. 2016 [19]. **Table S10.** Differentially expressed proteins/transcripts found in the integrative analysis that were also differentially expressed in EAB treatment vs control (TvC) experiment from Lane et al. 2016 [19]. **Table S11.** Differentially expressed transcripts found in the high-low and high-medium comparison that were also identified as EAB resistance genes by Kelly at al. 2020.

**Additional file 5: Table S12.** Location and sites of sampled trees. EAB-infested trees from five urban park sites located in the municipalities of Laval, Repentigny, Lavaltrie and Berthierville (Quebec, Canada). The GPS coordinates of each tree, the distance between trees at each site and the distances between sites are shown in this table.

## Data Availability

Transcriptomics data generated in this study (raw Illumina sequencing reads and processed data files) have been deposited in NCBI's Gene Expression Omnibus and are accessible through GEO Series accession number GSE212332. The proteomics data (MS raw files and MaxQuant search results) have been deposited on ProteomeXchange (PXD037126) through MassIVE repository.

## Abbreviations

EAB: Emerald ash borer
DNA: Deoxyribonucleic acid
GO: Gene ontology
HCD: Higher energy collision induced dissociation
INRS: Institut national de la recherche scientific
LC-MSMS: Liquid chromatography coupled to tandem mass spectrometry
LFQ: Label free quantification
MS: Mass spectrometry
mRNA: Messenger ribonucleic acid
NCBI: National center for biotechnology information
PCR: Polymerase chain reaction
qPCR: Quantitative polymerase chain reaction
RNA-seq: Ribonucleic acid sequencing
sPLS-DA: Sparse partial-least-squares discriminant analysis
