# Supplementary material for: Integrative analysis of green ash phloem transcripts and proteins during an emerald ash borer infestation
Source: BMC Plant Biol. 2023 Mar 3;23:123. doi: 10.1186/s12870-023-04108-y (PMC9983263; doi:10.1186/s12870-023-04108-y)
Supplement: Supplementary file 1 — Additional file 1: Figure S1. qPCR validation of RNA seq experiment. Normalized counts of RNA-Seq (blue) compared to Fold change of qPCR results (yellow) of eleven selected genes. [file 12870_2023_4108_MOESM1_ESM.pdf]

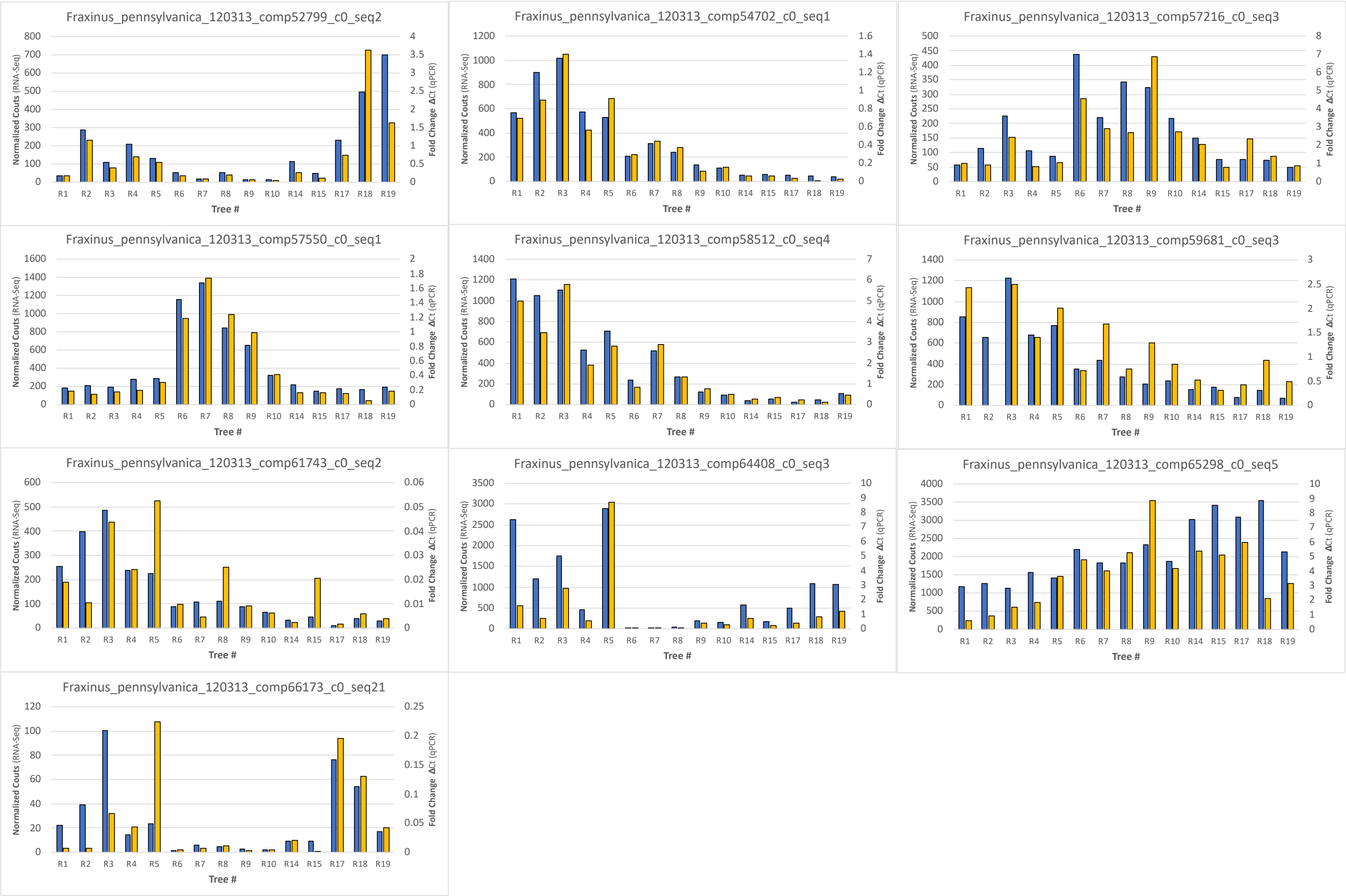

Figure S1. qPCR validation of RNA seq experiment. Normalized counts of RNA-Seq (blue) compared to Fold change of qPCR results (yellow) of eleven selected genes.
