# Supplementary material for: Integrative analysis of green ash phloem transcripts and proteins during an emerald ash borer infestation
Source: BMC Plant Biol. 2023 Mar 3;23:123. doi: 10.1186/s12870-023-04108-y (PMC9983263; doi:10.1186/s12870-023-04108-y)
Supplement: Supplementary file 2 — Additional file 2: Figure S2. Corelation of qPCR validation to RNA seq experiment. Normalize counts of RNA-Seq compared to Fold change of qPCR results of eleven selected genes. R value is shown on each chart. [file 12870_2023_4108_MOESM2_ESM.pdf]

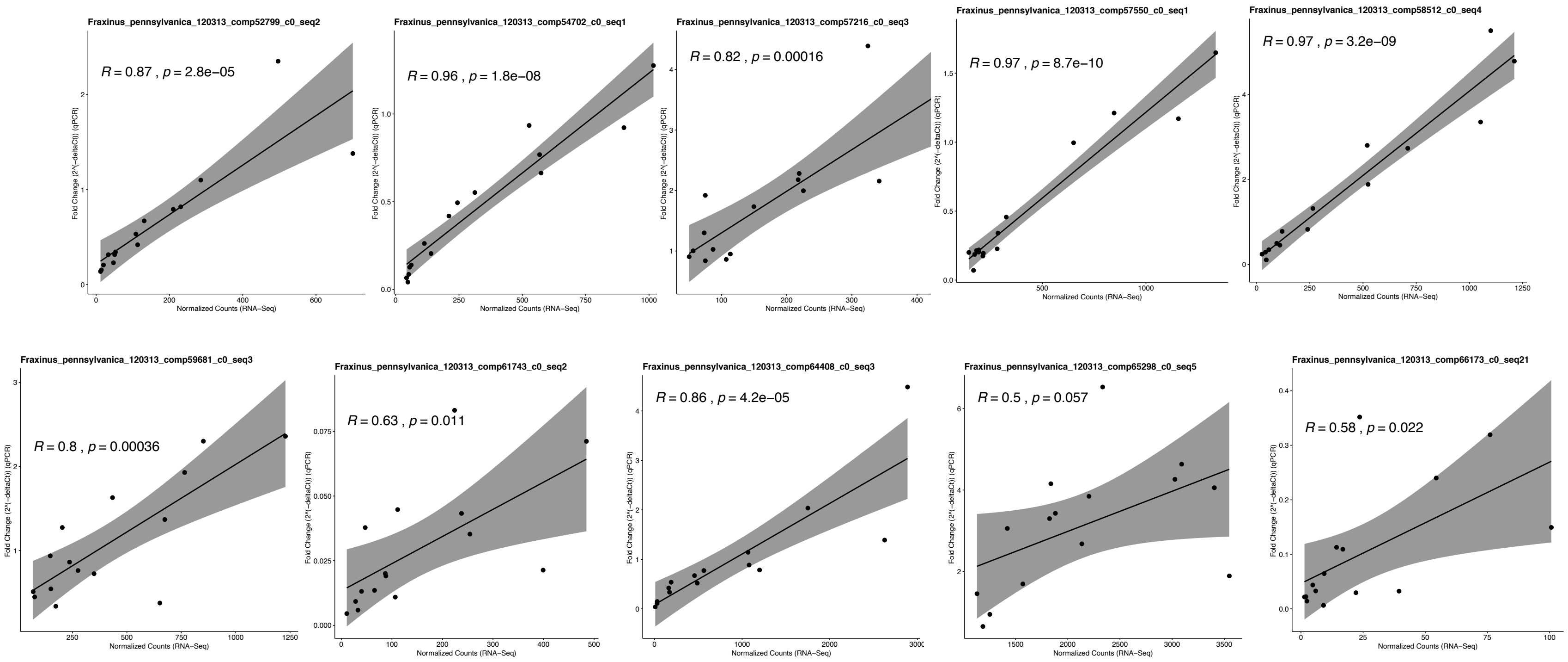

Figure S2. Corelation of qPCR validation to RNA seq experiment. Normalize counts of RNA-Seq compared to Fold change of qPCR results of eleven selected genes. R value is shown on each chart.
