# Supplementary material for: Integrative analysis of green ash phloem transcripts and proteins during an emerald ash borer infestation
Source: BMC Plant Biol. 2023 Mar 3;23:123. doi: 10.1186/s12870-023-04108-y (PMC9983263; doi:10.1186/s12870-023-04108-y)
Supplement: Supplementary file 3 — Additional file 3: Table S1. Total trimmed reads mapped to F. pennsylvanica transcriptome. [file 12870_2023_4108_MOESM3_ESM.pdf]

Table S1. Total trimmed reads mapped to *F. pennsylvanica* transcriptome

Tree #	Infestation Category	Total trimmed reads	Mapped in pairs		Mapped in broken pairs		Not mapped	
**1**	High	14 690 612	11 708 958	80%	1 810 880	12%	1 170 774	8%
**2**	High	15 512 710	12 822 416	83%	1 739 559	11%	950 735	6%
**3**	High	22 050 742	17 426 402	79%	2 698 548	12%	1 925 792	9%
**4**	High	13 189 032	10 545 722	80%	1 496 291	11%	1 147 019	9%
**5**	High	21 974 406	17 670 404	80%	2 499 590	11%	1 804 412	8%
**6**	Medium	12 432 224	9 376 926	75%	1 989 450	16%	1 065 848	9%
**7**	Medium	22 925 036	17 923 634	78%	2 981 272	13%	2 020 130	9%
**8**	Medium	22 347 330	16 101 116	72%	4 013 535	18%	2 232 679	10%
**9**	Medium	24 771 408	18 770 468	76%	3 405 300	14%	2 595 640	10%
**10**	Medium	20 889 444	16 925 668	81%	2 276 877	11%	1 686 899	8%
**14**	Low	20 812 264	16 225 350	78%	2 888 410	14%	1 698 504	8%
**15**	Low	19 691 518	15 287 428	78%	2 783 466	14%	1 620 624	8%
**17**	Low	22 330 642	17 657 602	79%	2 920 052	13%	1 752 988	8%
**18**	Low	23 646 162	17 832 480	75%	3 817 122	16%	1 996 560	8%
**19**	Low	19 914 928	15 635 408	79%	2 695 717	14%	1 583 803	8%
