# Supplementary material for: Integrative analysis of green ash phloem transcripts and proteins during an emerald ash borer infestation
Source: BMC Plant Biol. 2023 Mar 3;23:123. doi: 10.1186/s12870-023-04108-y (PMC9983263; doi:10.1186/s12870-023-04108-y)
Supplement: Supplementary file 5 — Additional file 5: Table S12. Location and sites of sampled trees. EAB-infested trees from five urban park sites located in the municipalities of Laval, Repentigny, Lavaltrie and Berthierville (Quebec, Canada). The GPS coordinates of each tree, the distance between trees at each site and the distances between sites are shown in this table. [file 12870_2023_4108_MOESM5_ESM.pdf]

Table S12. Location and sites of sampled trees. EAB-infested trees from five urban park sites located in the municipalities of Laval, Repentigny, Lavaltrie and Berthierville (Quebec, Canada). The GPS coordinates of each tree, the distance between trees at each site and the distances between sites are shown in this table.

Tree #	Infestation Category	Location	Site	GPS coordinates	Distance between trees (meters)	Distance between sites (kilometers)
**1**	High	Laval	INRS	45.54118362000974, -73.7191003887428	1 and 2: 31 m<br>1 and 3: 36 m	Laval and île Lebel: 31 km<br>Laval and Parc Entramis: 31 km<br>Laval and Parc Gérard Lavallée: 51 km<br>Laval and Parc Chapelle des Cuthbert: 74 km
**2**	High	Laval	INRS	45.54157681546907, -73.71915411045124	2 and 3: 6 m<br>2 and 4: 21 m	
**3**	High	Laval	INRS	45.541586222025316, -73.71914873828041	3 and 4: 24 m<br>3 and 5: 88 m	
**4**	High	Laval	INRS	45.54146057764722, -73.71937008533232	1 and 4: 19 m<br>4 and 5: 73 m	
**5**	High	Laval	INRS	45.54080898026276, -73.71916573238266	1 and 5: 57 m<br>2 and 5: 78 m	
**6**	Medium	Repentigny	île Lebel	45.73828486932364, -73.44156945111312	6 and 7: 36 m	île Lebel and Parc Entramis: 2.5 km<br>île Lebel and Gérard Lavallée: 21 km<br>île Lebel and Chapelle des Cuthbert: 44 km
**7**	Medium	Repentigny	île Lebel	45.73856466634799, -73.44131269968666		
**8**	Medium	Repentigny	île Lebel	45.73668779882954, -73.44361445365824	6 and 8: 232 m	
**9**	Medium	Repentigny	Parc Entramis	45.754099095792434, -73.461038481575	9 and 10: 57 m	Parc Entramis and Parc Gérard Lavallée: 20 km<br>Parc Entramis and Parc Chapelle des Cuthbert: 43 km
**10**	Medium	Repentigny	Parc Entramis	45.75641823040004, -73.46071125066224		
**14**	Low	Lavaltrie	Parc Gérard Lavallée	45.88360959140864, -73.27600928468352	14 and 15: 75 m	Parc Gérard Lavallée and Chapelle des Cuthbert: 24 km
**15**	Low	Lavaltrie	Parc Gérard Lavallée	45.88360210126801, -73.27541211199188		
**17**	Low	Berthierville	Chapelle des Cuthbert	46.08980294719966, -73.17693768071061	17 and 18: 22 m	
**18**	Low	Berthierville	Chapelle des Cuthbert	46.08966529275788, -73.17703424024975	18 and 19: 46 m	
**19**	Low	Berthierville	Chapelle des Cuthbert	46.089189080147214, -73.17685721442798	17 and 19: 68 m	
